# Efficacy and Safety of Self-Expandable Covered Metallic Stents for Benign and Malignant Ureteral Obstructions: A Long-Term Retrospective Study

**DOI:** 10.3390/medicina61020351

**Published:** 2025-02-17

**Authors:** Sae Woong Choi, Yong Sun Choi, Kang Sup Kim, Hyuk Jin Cho

**Affiliations:** 1 Department of Urology, Yeouido St. Mary’s Hospital, College of Medicine, The Catholic University of Korea, Seoul 07345, Republic of Korea; dextral@nate.com; 2 Department of Urology, Eunpyeong St. Mary’s Hospital, College of Medicine, The Catholic University of Korea, Seoul 03312, Republic of Korea; yschoi1008@catholic.ac.kr; 3 Department of Urology, Uijeongbu St. Mary’s Hospital, College of Medicine, The Catholic University of Korea, Uijeongbu 11765, Republic of Korea; prodigy81@catholic.ac.kr; 4 Department of Urology, Seoul St. Mary’s Hospital, College of Medicine, The Catholic University of Korea, Seoul 06591, Republic of Korea

**Keywords:** ureteral obstruction, stents, interventional procedure, ureter stricture

## Abstract

*Background and Objectives:* This study evaluated the safety and efficacy of long-term indwelling self-expandable covered metallic stents (UVENTA; Taewoong Medical Co., Ltd., Seoul, Republic of Korea) used to manage benign and malignant ureteral strictures. *Materials and Methods:* We retrospectively identified and analyzed the medical records of all patients who underwent metallic stent insertion at our institution since September 2012. Additionally, we evaluated the technical and clinical success rates and complications of patients who underwent follow-up for more than 36 months. *Results:* A total of 25 patients underwent metallic stent insertion for ureteral obstructions at our institution. Among them, 18 underwent follow-up for more than 36 months. A total of 21 ureters (15 unilateral and 3 bilateral) were ultimately included in this study. Metallic stents were successfully placed in all ureters using a retrograde approach, with a technical success rate of 100%. The mean follow-up duration was 58.6 months (range, 36–107 months). However, the clinical success rates were 85.7% (18/21 ureters) by 12 months, 61.9% (14/21 ureters) by 24 months, and 52.4% (11/21 ureters) after 36 months. During follow-up, obstructions could not be resolved using metallic stents in eleven ureters (median time to failure, 18.4 months; range, 2−40 months); therefore, they were treated with nephrectomy (three ureters because of a nonfunctional kidney) or percutaneous nephrostomy and double J stent placement (four ureters). Major complications included the encrustation of the metallic stent, flank pain, and gross hematuria. A uretero-enteric fistula occurred in one ureter. In two patients, existing metallic stents were removed and patency was maintained. In another two patients, new metallic stents were inserted without complications. *Conclusions:* Benign and malignant ureteral obstructions may be treated effectively and safely with metallic stents. However, the patency rate drastically decreased and major complications occurred during long-term follow-up. Therefore, careful patient selection is necessary to achieve better results.

## 1. Introduction

Benign and malignant ureteral obstructions are generally managed by inserting double J (DJ) ureteral stents or performing percutaneous nephrostomy (PCN). Although these modalities are easy to perform for indwelling stents and immediately relieve obstruction in most patients, they can lead to many adverse side effects [1]. DJ ureteral stents are commonly associated with complications such as the irritation of the lower urinary tract, hematuria, urinary tract infections, reflux, migration, and encrustation [2,3,4]. In particular, periodic exchange of DJ ureteral stents can result in limited patency, which is an important drawback. PCN involves the use of an external tube and significant discomfort, resulting in a lower quality of life. The ideal ureteral stent should not cause symptoms, should be sufficiently durable to ensure long-term use, and should provide normal drainage without encrustation. Several metallic stents such as the Resonance stent (Cook Medical, Bloomington, IN, USA), Silhouette stent (Applied Medical, Rancho Santa Margarita, CA, USA), Memokath 051 stent (PNN Medical A/S, Kvistgaard, Denmark), Allium stent (Allium Medical Solutions Ltd., Caesarea, Israel), and UVENTA stent (Taewoong Medical Co., Ltd., Seoul, Republic of Korea) have been introduced and used to relieve obstructions.

Since Kim et al. [5] reported their initial experiences with metallic stents, novel self-expandable covered metallic stents have become an available option in Korea for relieving benign and malignant obstructions. Some initial studies of the use of metallic stents for the treatment of ureteral obstructions have reported excellent results without severe complications [6,7]. However, because only two studies have reported the long-term follow-up results of metallic stents, the published data are limited [8,9]. The respective follow-up durations in these two studies were 30.9 and 34.9 months. We thought that it would be helpful to conduct a study with a follow-up period of more than 36 months to confirm the safety and effectiveness of the self-expandable covered metallic stent. Therefore, the aim of the present study was to assess the long-term efficacy, safety, and patency of metallic stents used to treat benign and malignant ureteral obstructions for more than 36 months.

## 2. Materials and Methods

After obtaining approval from the Institutional Review Board (IRB) of our hospital (IRB approval number KC18RESI0766), all procedures were conducted in accordance with the ethical guidelines of the Declaration of Helsinki and its later amendments or comparable ethical standards.

### 2.1. Inclusion and Exclusion Criteria

The inclusion criteria were clinically diagnosed benign or malignant ureteral obstruction and age older than 20 years. The exclusion criteria were uncontrolled acute and chronic infections or the inflammation of the genitourinary tract, severe gross hematuria that made it difficult to view the bladder during cystoscopy, and patients with severe systemic diseases who were not suitable candidates for surgery and anesthesia.

### 2.2. Study Design, Collected Data, and Indication

We performed a retrospective review of patients who had retained metallic stents for a minimum of 36 months at our institution’s Department of Urology between September 2012 and May 2015. Of the 25 patients with indwelling metallic stents (31 ureters) identified during the study period, 18 patients (21 ureters) met the inclusion criterion of at least 36 months of follow-up without loss. The electronic medical records and prospectively maintained data regarding metallic stents were searched to acquire follow-up data. Demographic characteristics including age, sex, side, etiology, stricture site, and complications as well as stent-related symptoms, surgical parameters, complications, and results were collected.

All patients had DJ stents placed prior to the insertion of metallic stents. Metallic stents were inserted in patients who desired alternative treatment options due to complications or discomfort associated with DJ stents, or who experienced the persistent malfunction of DJ stents. During the study period, the primary reasons for metallic stent placement were severe irritative voiding symptoms caused by DJ stents (47.6%, 10/21), the inconvenience of frequent DJ stent changes (38.1%, 8/21), and the malfunction of DJ stents (14.3%, 3/21).

### 2.3. Surgical Procedure

An experienced endourological urologist at our hospital performed metallic stent placement. The details of metallic stent insertion were described previously [5,7]. Briefly, under general anesthesia, we conducted retrograde pyelography to confirm the stricture site, level, and length before metallic stent insertion. All metallic stents were inserted using a retrograde approach under cystoscopic and fluoroscopic guidance. A stiff guidewire was placed to maintain strength during balloon dilation and stent placement. The metallic stent expanded spontaneously because of the original radial force. If the metallic stent did not expand completely after insertion, then additional ureteral balloon dilation was performed using a 6 mm balloon dilation catheter (UroMax Ultra; Boston Scientific, Marlborough, MA, USA). For cases involving a long ureteral stricture, additional stents were placed with an overlap of 2 to 3 cm.

### 2.4. Follow-Up and Outcome Measures

All patients were followed up with at the outpatient clinic every 3 months after stent insertion. During follow-up, serum laboratory tests, urinalysis, urine cultures, intravenous urography or computed tomography, and diuretic renography, if necessary, were performed. The patency of UVENTA stents and stent-related complications such as flank pain, severe gross hematuria, and fever were evaluated and investigated during every outpatient visit. During a previous study, we evaluated the technical and clinical success and failure rates of metallic stents to assess their efficacy [7]. Successful stent insertion in the ureteral stricture without immediate complications was confirmed by intraoperative fluoroscopy and perioperative radiography and defined as technical success. Clinical success was defined as the amelioration of renal function and improved or no hydronephrosis on intravenous urography, kidney sonography, or computed tomography. Failure was defined as stent-related complications such as stent migration, premature removal, the retention of other ureteral stents, and intolerance to stent-related symptoms. Success was evaluated every 3 months. Stent-related complications were classified according to the modified Clavien–Dindo classification system. Major complications were defined as those requiring additional surgical, endoscopic, or radiologic procedures under general anesthesia.

## 3. Results

During the study period, 30 patients met the inclusion criteria. Two patients were excluded due to septic shock complicated by urolithiasis, and one due to concurrent bladder cancer, and two others were excluded due to severe cardiopulmonary conditions that contraindicated general anesthesia. Ultimately, 25 patients underwent metallic stent insertion for ureteral obstruction at our institution. Among them, 18 underwent follow-up for more than 36 months. A total of 21 ureters (15 unilateral and 3 bilateral) were included in this study (Table 1). The median age was 57.5 years (range, 29–82 years). In 13 ureters, obstruction was caused by malignancy; however, in eight, obstruction was caused by benign diseases. The main reasons for using metallic stents were the inconvenience of regular DJ stent changes within short time periods (8 ureters), severe lower urinary tract symptoms caused by DJ stents (10 ureters), and persistent obstructions (3 ureters).

All metallic stents were successfully placed and correctly positioned in all 21 ureters. Therefore, the technical success rate was 100% (21/21 ureters). No procedure-related complications were observed. The median follow-up duration was 58.6 months (range, 36–107 months). The success rates were 85.7% (18/21 ureters) by 12 months, 61.9% (14/21 ureters) by 24 months, and 52.4% (11/21 ureters) after 36 months. The average duration until treatment failure was 18.4 months (range, 2–40 months). The reasons for metallic stent failure included stent migration in two ureters, stent malfunction because of hyperplasia or encrustation in four ureters, a uretero-enteric fistula in one ureter after metallic stent insertion, and persistent irritation, pain, or hematuria in one ureter (Table 2). Two of the four cases with stent malfunction experienced urinary tract infections, and the case with a uretero-enteric fistula also developed an infection. In two patients, existing metallic stents were removed and patency was maintained post-procedure. In two other patients, with minimal encrustation and no complications, new metallic stents were placed to prevent obstruction.

Table 2 presents the changes in renal function according to treatment success. While there was a tendency for serum creatinine levels to increase more in the failure group during the follow-up period in patients with metallic stents, this difference did not reach statistical significance (*p*-value = 0.072).

The major complications associated with metallic stents are summarized in Table 3. Four cases of complete stent obstruction were observed. The causes of obstruction included stone encrustation and mucosal hyperplasia. Stent removal using endoscopic procedures is difficult because of the severe adhesion of the metallic stent to the ureteral tissue; furthermore, the affected kidneys were nonfunctional. Therefore, laparoscopic simple nephrectomy was recommended for all cases. One patient experienced a direct ureteral injury with fistula formation in the small intestine and underwent ureteral ligation with PCN instead of the simultaneous reconstruction of the ileal ureter.

## 4. Discussion

Ureteral obstruction is a common finding that is considered a troublesome challenge for urologists [10]. Specifically, proper long-term upper urinary tract drainage in patients with malignant or benign ureteral obstructions is a therapeutic challenge. Hydronephrosis reduction is necessary to maintain renal function and prevent urinary tract infections such as sepsis, thus improving the quality of life [11]. Currently, various therapeutic options for ureteral obstructions exist, such as DJ ureteral stent placement, PCN, endoureterostomy, ureteral balloon dilatation, and surgical reconstruction [12,13]. Treatment must be determined according to the etiology and severity of the ureteral obstruction, complications, and prognosis [14]. DJ stent placement and PCN are relatively safe and easy to perform and result in immediate renal decompression and function recovery. DJ ureteral stent placement and PCN have advantages; therefore, the placement of DJ ureteral stents using balloon dilation has become a popular procedure for the treatment of ureteral obstructions [15]. However, the success rate of DJ ureteral stent placement is not high [16,17]. Furthermore, DJ ureteral stents are inconvenient because they must be changed every 3 months [18]. Additionally, they are vulnerable to occlusion caused by extrinsic compression [19]. These disadvantages of DJ ureteral stent placement and PCN negatively affect the quality of life. Metallic stents have been introduced as a less invasive and more long-term treatment without frequent stent changes, PCN, and irritating voiding symptoms. Patients who wish to avoid PCN or frequent stent changes are also good candidates for metallic stent placement. Various types of metallic stents have been developed, each with its own benefits and limitations [17].

Pauer et al. [20] first reported the use of metallic stents for 12 patients with malignant ureteral strictures. Subsequently, another study reported 10 patients who received self-expandable stents and experienced excellent results in terms of patency after 1 year [21]. Since then, various types of metallic stents have been developed and adopted. However, selecting the best metallic stent for a specific circumstance is difficult because each one has a different mechanism. UVENTA stents consist of a triple-layer mesh structure, an inner mesh with a polytetrafluoroethylene membrane that prevents tissue ingrowth, and an outer mesh with a friction coefficient that prohibits stent migration [7]. Different stent sizes and different plasticity levels of the stents allow better adaptation to the ureteral obstruction.

Studies of UVENTA stents have revealed promising results in terms of patency, migration, and complications such as hematuria, infection, encrustation, and pain during short-term follow-up [5,6,22,23]. Chung et al. [6] reported a primary stent success rate of 64.8% and an overall success rate of 81.7% for 71 ureters. We believe that this high success rate was attributable to the relatively short follow-up duration (mean, 11 months). Additionally, they reported that the most common cause of obstruction was tumor progression beyond the ureteric segment treated with a metallic stent. To our knowledge, the present study is the longest follow-up study to evaluate the effectiveness and safety of metallic stents for the management of malignant and benign ureteral obstructions. Therefore, this study provides important information regarding the natural history of malignant and benign obstructions in ureters with metallic stents. Furthermore, this study revealed a technical success rate of 100% and metallic stent patency rate of 52.4% during a median follow-up duration of more than 24 months. We performed metallic stent insertion using a retrograde approach. Retrograde metallic stent placement offers several advantages over antegrade stent insertion. For example, it does not require nephrostomy; therefore, there are no potential risks related to percutaneous access such as internal solid organ injury, pleural complications, and severe bleeding. Retrograde placement enables endoscopic procedures; therefore, concomitant procedures including diagnostic endoscopy, abnormal uroepithelial tissue biopsies, and stent removal are possible. Furthermore, when stent placement is not performed at the desired location, the distal stent position can be easily adjusted using retrograde procedures. Most importantly, the majority of urologists are familiar with retrograde metallic stent placement. In the present study, severe complications occurred in four patients. Only two previous studies have reported severe metallic stent-related complications [8,24]. These inconsistencies are likely attributable to the short follow-up periods of those previous studies. Kim et al. [8] reported uretero-arterial fistulas (6%), uretero-enteric fistulas (6%), uretero-vaginal fistulas (2%), uncontrollable bleeding (2%), and stone encrustation (4%); additionally, they reported that the most common major complication was direct ureteral injury with or without fistula formation in nearby organs. They hypothesized that the multilayered structure of the metallic stent contributed to fistula formation. While designed to provide radial strength for patency and withstand ureteral compression, the stent's structure was presumed to have played a role in the development of uroepithelial ischemia and subsequent ureteral injury. Their study included three uretero-arterial fistulas and life-threatening complications, which were managed with endovascular stenting and delayed open surgery. During our study, complete stent obstruction occurred in three patients. Metallic stents are designed to withstand stent migration and uroepithelial tissue ingrowth. Nonetheless, functional failure of metallic stents and stone formation were unavoidable during long-term follow-up. Furthermore, these completely obstructed metallic stents could not be removed using cystoscopic procedures and renal function was very low. Therefore, these three patients underwent laparoscopic simple nephrectomy. A uretero-enteric fistula occurred in one patient; therefore, primary bowel repair and ureteral ligation with PCN were performed. Song et al. [24] reported the pathogenesis of uretero-enteric fistulas. Previous pelvic surgery and radiation therapy are typical risk factors for uretero-enteric fistulas, and balloon dilatation and a history of abdominal surgeries that can induce severe adhesion between the bowel and ureter are additional risk factors for uretero-enteric fistulas. Metallic stents are mainly used to avoid frequent stent changes and attain long-term or permanent indwelling. However, when patients experience complications such as stent malfunction, migration, recurrent urinary tract infections, and intractable stent-induced pain, the metallic ureteral stents should be removed. Therefore, feasible stent removal or exchange methods are necessary.

Surprisingly, in two patients, patency was maintained even after the metallic stents were removed without complications in this study. This may have occurred because passive dilatation was achieved using a metallic stent. Therefore, when patency is expected to be maintained, removal of the metallic stents after indwelling for a certain period is a good treatment option when they are not functioning appropriately.

This retrospective study had some limitations. First, the sample size was small because DJ stents and PCN are mainly used for ureteral obstructions; metallic stents are rarely used. Second, we could not compare the long-term effectiveness and safety of metallic and DJ stents. Furthermore, we also notice that very few functional studies such as radionuclide scans were performed. We did not examine quality of life changes and stent-related symptoms and by using validated questionnaires. It is mandatory to determine whether there are any merits of UVENTA stents over DJ stents by performing a comparative study. Lastly, stent-related urinary symptoms were not evaluated using questionnaires. However, this study represents the longest follow-up of UVENTA stents to date, providing valuable clinical insights into the success rates, failure modes, and complications associated with long-term metallic stent placement.

## 5. Conclusions

This retrospective study assessed the long-term efficacy and safety of UVENTA metallic stents in managing benign and malignant ureteral obstructions. While demonstrating initial technical success and reasonable short-term patency, the long-term outcomes revealed significant limitations. Despite their potential as an alternative treatment for ureteral obstructions, the long-term efficacy and safety of metallic stents require further investigation and refinement. Therefore, careful patient selection is necessary to achieve better results.

## Figures and Tables

**Table 1:** Demographic characteristics of the patients.

Variables	Overall
Patients, n	18
Ureters, n	21
Age, years, median (range)	57.5 (29–82)
Sex, male/female, n	8/10
Side, n	
Right	10
Left	5
Bilateral	3
Stricture location, n	
Proximal	3
Middle	5
Distal	4
Multiple	9
Stricture etiology, n	
Malignancy (the direct or nodal compression of the ureter)	13
Colorectal cancer	3
Stomach cancer	2
Gynecological cancer	8
Benign	8
Idiopathic	5
Retroperitoneal fibrosis	2
Benign ureteral mass	1
Prior treatment, n	
Double J stent	19
Percutaneous nephrostomy	2

**Table 2:** Long-term treatment outcomes of metallic stents.

Outcomes		Total (n = 21)
1. Follow-up duration, months		58.6 (36–107)
2. Success rate, n		
12 months		18 (85.7%)
24 months		14 (61.9%)
>36 months		11 (52.4%)
3. Time to failure, months		18.4 (2–40)
4. Cause of failure, n		
Stent migration		2
Stent malfunction because of hyperplasia or encrustation		4
Persistent irritation, pain, or hematuria		2
Uretero-enteric fistula		1
Recurrent urinary tract infection		1
5. Change in serum creatinine (mg/dL)		*p*-value, 0.072
Success > 36 months		
Pre, 1.13 ± 0.47	Post, 0.95 ± 0.35	0.18 ± 0.20
Failure		
Pre, 1.02 ± 0.43	Post, 1.12 ± 0.39	−0.1 ± 0.41

Pre, pre-procedure creatinine; Post, post-procedure creatinine.

**Table 3:** Major complications related to metallic stents.

Patient	Sex	Age (Years)	Stricture Site	Complication	Time to Cx (Months)	Treatment
1	F	36	Lower	Complete stent obstruction	39	Simple nephrectomy
2	F	59	Multiple	Uretero-enteric fistula	29	Ureteral ligation with PCN
3	F	29	Middle	Complete stent obstruction	38	Simple nephrectomy
4	M	49	Multiple	Complete stent obstruction	40	Simple nephrectomy

Cx, complication; F, female; M, male; PCN, percutaneous nephrostomy.

## Data Availability

The data presented in this study are available in the article. Further inquiries can be directed to the corresponding author.

## References

[1] Giannese D., Moriconi D., Cupisti A., Zucchi A., Pastore A.L., Simonato A., Mogorovich A., Claps F., Bartoletti R. (2023). Idiopathic Retroperitoneal Fibrosis: What Is the Optimal Clinical Approach for Long-Term Preservation of Renal Function?. Urol. Int..

[2] Zisman A., Siegel Y.I., Siegmann A., Lindner A. (1995). Spontaneous ureteral stent fragmentation. J. Urol..

[3] Culkin D.J., Zitman R., Bundrick W., Goel Y., Price V., Ledbetter S., Mata J.A., Venable D.D. (1992). Anatomic, functional, and pathologic changes from internal ureteral stent placement. Urology.

[4] Docimo S.G., Dewolf W.C. (1989). High failure rate of indwelling ureteral stents in patients with extrinsic obstruction: Experience at 2 institutions. J. Urol..

[5] Kim J.H., Song K., Jo M.K., Park J.W. (2012). Palliative care of malignant ureteral obstruction with polytetrafluoroethylene membrane-covered self-expandable metallic stents: Initial experience. Korean J. Urol..

[6] Chung K.J., Park B.H., Park B., Lee J.H., Kim W.J., Baek M., Han D.H. (2013). Efficacy and safety of a novel, double-layered, coated, self-expandable metallic mesh stent (Uventa™) in malignant ureteral obstructions. J. Endourol..

[7] Kim K.S., Choi S., Choi Y.S., Bae W.J., Hong S.-H., Lee J.Y., Kim S.W., Hwang T.-K., Cho H.J. (2014). Comparison of efficacy and safety between a segmental thermo-expandable metal alloy spiral stent (Memokath 051) and a self-expandable covered metallic stent (UVENTA) in the management of ureteral obstructions. J. Laparoendosc. Adv. Surg. Tech..

[8] Kim M., Hong B., Park H.K. (2016). Long-Term Outcomes of Double-Layered Polytetrafluoroethylene Membrane-Covered Self-Expandable Segmental Metallic Stents (Uventa) in Patients with Chronic Ureteral Obstructions: Is It Really Safe?. J. Endourol..

[9] Choi J., Chung K.J., Choo S.H., Han D.H. (2019). Long-term outcomes of two types of metal stent for chronic benign ureteral strictures. BMC Urol..

[10] López-Huertas H.L., Polcari A.J., Acosta-Miranda A., Turk T.M. (2010). Metallic ureteral stents: A cost-effective method of managing benign upper tract obstruction. J. Endourol..

[11] Rodríguez A.R., Lockhart A., King J., Wiegand L., Carrion R., Ordorica R., Lockhart J. (2011). Cutaneous ureterostomy technique for adults and effects of ureteral stenting: An alternative to the ileal conduit. J. Urol..

[12] Kachrilas S., Bourdoumis A., Karaolides T., Nikitopoulou S., Papadopoulos G., Buchholz N., Masood J. (2013). Current status of minimally invasive endoscopic management of ureteric strictures. Ther. Adv. Urol..

[13] Lucas J.W., Ghiraldi E., Ellis J., Friedlander J.I. (2018). Endoscopic Management of Ureteral Strictures: An Update. Curr. Urol. Rep..

[14] Ku J.H., Lee S.W., Jeon H.G., Kim H.H., Oh S.J. (2004). Percutaneous nephrostomy versus indwelling ureteral stents in the management of extrinsic ureteral obstruction in advanced malignancies: Are there differences?. Urology.

[15] Kim M., Song G., Park S.H., Sohn M., Song S.H., Park H.K., Hong B. (2016). Outcomes of patients with ureteral obstruction who achieved stent-free state following balloon dilatation. Scand. J. Urol..

[16] Ganatra A.M., Loughlin K.R. (2005). The management of malignant ureteral obstruction treated with ureteral stents. J. Urol..

[17] Yossepowitch O., Lifshitz D.A., Dekel Y., Gross M., Keidar D.M., Neuman M., Livne P.M., Baniel J. (2001). Predicting the success of retrograde stenting for managing ureteral obstruction. J. Urol..

[18] Haifler M., Shvero A., Zilberman D., Ramon J., Winkler H.Z., Margel D., Kleinmann N. (2020). Tandem Ureteral Stents for Malignant Ureteral Obstruction. J. Endourol..

[19] Chung S.Y., Stein R.J., Landsittel D., Davies B.J., Cuellar D.C., Hrebinko R.L., Tarin T., Averch T.D. (2004). 15-year experience with the management of extrinsic ureteral obstruction with indwelling ureteral stents. J. Urol..

[20] Pauer W., Lugmayr H. (1992). Metallic Wallstents: A new therapy for extrinsic ureteral obstruction. J. Urol..

[21] Flueckiger F., Lammer J., Klein G.E., Hausegger K., Lederer A., Szolar D., Tamussino K. (1993). Malignant ureteral obstruction: Preliminary results of treatment with metallic self-expandable stents. Radiology.

[22] Kim K.H., Cho K.S., Ham W.S., Hong S.J., Han K.S. (2015). Early Application of Permanent Metallic Mesh Stent in Substitution for Temporary Polymeric Ureteral Stent Reduces Unnecessary Ureteral Procedures in Patients With Malignant Ureteral Obstruction. Urology.

[23] Chung H.H., Kim M.D., Won J.Y., Won J.H., Cho S.B., Seo T.S., Park S.W., Kang B.C. (2014). Multicenter experience of the newly designed covered metallic ureteral stent for malignant ureteral occlusion: Comparison with double J stent insertion. Cardiovasc. Interv. Radiol..

[24] Song G., Lim B., Han K.S., Song S.H., Park H.K., Hong B. (2015). Complications after polymeric and metallic ureteral stent placements including three types of fistula. J. Endourol..

